# Can Occupancy–Abundance Models Be Used to Monitor Wolf Abundance?

**DOI:** 10.1371/journal.pone.0102982

**Published:** 2014-07-23

**Authors:** M. Cecilia Latham, A. David M. Latham, Nathan F. Webb, Nicole A. Mccutchen, Stan Boutin

**Affiliations:** 1 Landcare Research, Lincoln, Canterbury, New Zealand; 2 Alberta Environment and Sustainable Resource Development, Edmonton, Alberta, Canada; 3 Department of Environment and Natural Resources, Government of the Northwest Territories, Yellowknife, Northwest Territories, Canada; 4 Department of Biological Sciences, University of Alberta, Edmonton, Alberta, Canada; Università degli Studi di Napoli Federico II, Italy

## Abstract

Estimating the abundance of wild carnivores is of foremost importance for conservation and management. However, given their elusive habits, direct observations of these animals are difficult to obtain, so abundance is more commonly estimated from sign surveys or radio-marked individuals. These methods can be costly and difficult, particularly in large areas with heavy forest cover. As an alternative, recent research has suggested that wolf abundance can be estimated from occupancy–abundance curves derived from “virtual” surveys of simulated wolf track networks. Although potentially more cost-effective, the utility of this approach hinges on its robustness to violations of its assumptions. We assessed the sensitivity of the occupancy–abundance approach to four assumptions: variation in wolf movement rates, changes in pack cohesion, presence of lone wolves, and size of survey units. Our simulations showed that occupancy rates and wolf pack abundances were biased high if track surveys were conducted when wolves made long compared to short movements, wolf packs were moving as multiple hunting units as opposed to a cohesive pack, and lone wolves were moving throughout the surveyed landscape. We also found that larger survey units (400 and 576 km^2^) were more robust to changes in these factors than smaller survey units (36 and 144 km^2^). However, occupancy rates derived from large survey units rapidly reached an asymptote at 100% occupancy, suggesting that these large units are inappropriate for areas with moderate to high wolf densities (>15 wolves/1,000 km^2^). Virtually-derived occupancy–abundance relationships can be a useful method for monitoring wolves and other elusive wildlife if applied within certain constraints, in particular biological knowledge of the surveyed species needs to be incorporated into the design of the occupancy surveys. Further, we suggest that the applicability of this method could be extended by directly incorporating some of its assumptions into the modelling framework.

## Introduction

Large mammalian carnivores provide conservation biologists and resource managers with complex challenges, such as endangered carnivore species recovery, human-carnivore conflict, and the management of carnivore species to conserve threatened and endangered prey. An inherent obstacle to the effective management of carnivores is the difficulty in obtaining reliable density estimates [1], [2]. Wolves (*Canis lupus*) are an excellent example because they are wide-ranging habitat generalists, exist at low densities, and are often secretive and reclusive, making surveys that rely on direct sightings difficult or impossible. Consequently, indirect measures of abundance (e.g., howling surveys, harvest statistics, catch-per-unit-effort, and hunter sightings) have often been used instead [3]–[5]. Wolf densities have, however, been estimated in many locations across North America and are becoming increasingly common in the literature (e.g., [6]). These estimates are usually obtained by intensive aerial snow-tracking or radiotelemetry [7], [8]. However, telemetry studies are expensive, requiring animal capture and radio-marking, and frequent monitoring of those individuals (usually over multiple years), making them impractical for broad regions. Further, in landscapes with ongoing wolf harvest seasons, it can be difficult to maintain an adequate sample of marked animals.

More cost-effective approaches such as transect intercept probability sampling and the sampling unit probability estimator (SUPE) have been used to estimate the density of wolves and other carnivore species [9]–[12]. These methods have proven to be useful in large study areas (≥10,000 km^2^) with open forest-cover types. However, the reliability of density estimates obtained using these methods and their cost-effectiveness are questionable in areas where track networks cannot be followed continuously from an aircraft due to heavy forest cover [12]. Accordingly, Webb and Merrill [13] proposed utilizing the relationship between occupancy and abundance [14]–[17] to estimate wolf abundance in areas where other approaches might not be feasible.

Occupancy–abundance relationships are a fundamental pattern in ecology [18]. Usually occupancy (the number of occupied survey units) increases with abundance and consequently researchers have applied this positive relationship to estimate the abundance of organisms via occupancy surveys [19]. However, as Webb and Merrill [13] note, the inherent difficulty of directly observing large carnivore species such as wolves remains a major obstacle and makes the utility of traditional occupancy–abundance models impractical. Accordingly, these authors suggested that if sampling could be standardized, an empirically-derived relationship between the proportion of survey units occupied by wolf tracks and abundance might provide a means to estimate wolf density. This would, however, require conducting occupancy surveys across multiple areas with known animal densities, which is likely unfeasible. As an alternative, Webb and Merrill [13] utilized movement models to derive an occupancy–abundance relationship from “virtual” surveys of simulated wolf track networks across a range of wolf pack densities. This technique has the potential to provide a time- and cost-effective approach to estimating wolf abundance across broad regions.

The virtually-derived occupancy–abundance relationship described above rests on a number of assumptions [13], related primarily to the biology of the surveyed species. Amongst these assumptions is that of similar movement rates among sampling intervals, cohesive social groups, and the ability of surveyors to distinguish wolf tracks made by lone animals. Furthermore, the occupancy-derived abundance estimates produced by this model are scale-specific and will vary with the size of the survey units [20]. For example, if a survey unit is too small, a greater proportion of units will be occupied if wolves make long versus short movements and thus abundance will be overestimated. Alternatively, if the survey unit size is too big, then the probability of detecting a species is always high [20], a result which provides little information when comparing across study areas or over time. Higher than expected rates of occupancy may also occur if the pack is not moving as a cohesive unit (e.g., pack members go on individual forays or are dispersing) and if lone wolves are moving throughout the study area (e.g., [21], [22]). Because these factors are likely to vary both within and among study areas, the utility of the virtually-derived occupancy–abundance approach hinges on the robustness of the method to changes in these factors.

In this study, we explored an approach similar to that of Webb and Merrill [13] to estimate wolf abundance using a movement-based occupancy–abundance model. Our primary objective was to determine how changing the size of the survey unit affects the sensitivity of the method to real-world conditions under which this model would be expected to apply, i.e. variation in wolf movement rates, changes in pack cohesion, and lone wolf movements. We compared occupancy–abundance relationships of 36 km^2^ grids with 144 km^2^, 400 km^2^, and 576 km^2^ grids. The smallest size corresponds with the size of the survey unit proposed by Webb and Merrill [13], the 144 km^2^ grid corresponds to the average daily distance moved by wolves in our area during winter (10 km), the 400 km^2^ grid corresponds to the 90th percentile of daily wolf movements during winter, and the largest size corresponds to the approx. size of the average winter wolf territory in our study area [8], [21].

## Methods

### Study Area

We evaluated the robustness of Webb and Merrill's [13] movement-based occupancy–abundance model using data from wolves in the western boreal plains in northeastern Alberta, Canada. Specifically, we used Global Positioning System (GPS) location data collected from wolves inhabiting the West Side of the Athabasca River (WSAR) and western portion of the East Side of the Athabasca River (ESAR) woodland caribou (*Rangifer tarandus caribou*) ranges. This area encompassed approx. 21,000 km^2^ of boreal mixed-wood and peatland vegetation within public lands near the town of Wabasca-Desmarais (55°57”N, 113°49”W).

Topographic relief within caribou range is minimal (500 m to 700 m), but increases to approx. 950 m in the Pelican Mountains (adjacent uplands) in the southwest of the study area. The Athabasca River, which flows south to north between WSAR and ESAR, is the lowest point at approx. 400 m. The study area is characterized by numerous other smaller rivers and streams. The study area is a naturally fragmented mosaic of peatlands (60%) and upland mixed-woods (40%). Peatlands consisted of black spruce (*Picea mariana*) bogs (60% of peatlands) and black spruce–tamarack (*Larix laricina*) fens (30% of peatlands). In addition, marshes and swamps interspersed peatlands throughout much of the study area. Well-drained upland mixed-woods were dominated by trembling aspen (*Populus tremuloides*), white spruce (*Picea glauca*), balsam fir (*Abies balsamea*), and jack pine (*Pinus banksiana*).

The study area has been extensively impacted by the energy sector, which consists of the creation of seismic lines (2–8 m wide) for exploration purposes and of well pads (1 ha), pipelines, and roads for oil and gas extraction purposes. Approximately 3.2% of the study area had been disturbed by the energy sector as of 2007 [23]. Because of the scarcity of merchantable timber in peatlands (i.e., caribou range), logging is generally confined to upland forests adjacent to the caribou ranges. Approximately 4.5% of the study area had been logged as of 2007.

### Ethics Statement

Our data collection complied with all relevant federal laws of Canada and provincial laws of Alberta. Capture and handling procedures adopted in this study were reviewed and approved by the University of Alberta Biosciences Animal Policy and Welfare Committee (Protocol No. 471503), and by the Government of Alberta (Alberta Environment and Resource Development Wildlife Research and Collection Permit Nos. 23428 and 23669).

### Wolf Data

We used location data from 11 GPS-collared wolves captured from 8 packs in January 2006–March 2008 (pack sizes: 2–22 wolves, average pack size  = 7.8) to derive parameters for movement-based occupancy–abundance models. We trapped wolves within WSAR and ESAR using modified foot-hold traps in summer and we caught wolves in winter via helicopter net-gunning. We fitted captured wolves with Lotek (Lotek Engineering, Newmarket, ON, Canada) 4400S (remote downloadable) collars programmed to obtain a location every 45 minutes in late-April to mid-June (i.e., wolf denning season) 2006 and every 2 hours for the remainder of our study. We only had 1 GPS-collared wolf per pack at any time. We differentially corrected GPS locations to reduce measurement error [24]. Previous trials in Alberta using Lotek GPS collars (with a high number of channels) on wolves have demonstrated minimal habitat-induced bias, suggesting that further corrections were unnecessary [25].

### Wolf movement-based occupancy modelling

The approach described in Webb and Merrill [13] involved dividing their study area into survey units, a sample of which were surveyed for the presence of wolves by simulating aerial searches for wolf tracks two days after fresh snowfall. The proportion of occupied survey units, as indicated by the presence of wolf tracks, provides an index of occupancy that can be converted to an actual wolf density estimate. The size of the survey unit used by Webb and Merrill [13] (36 km^2^) was equivalent to that used by the SUPE protocol [11], and was selected to ensure that the occupancy–abundance curve did not saturate at the high wolf pack densities present in their study area. To determine how changing survey unit size affects the sensitivity of this model to variation in wolf movement rates, changes in pack cohesion, and loner movements, we first created four regular survey grids using Geospatial Modelling Environment [26] in ArcGIS 10.1 (ESRI© Inc., USA). Each survey grid covered 14,400 km^2^ within WSAR (∼70% of the range) but differed in the size and number of survey units: (1) Grid 36 km^2^ had 400–6 km × 6 km survey units, (2) Grid 144 km^2^ had 100–12 km × 12 km survey units, (3) Grid 400 km^2^ had 36–20 km × 20 km survey units, and (4) Grid 576 km^2^ had 25–24 km × 24 km survey units.

We then constructed wolf territories for each of 12 wolf pack densities (0.0–5.5 packs/1000 km^2^) representing the range of observed densities across most of North America [6], including WSAR [8]. For each pack-density, wolf territories were represented by circular home ranges of sizes varying from 182 km^2^ (wolf pack density  = 5.5 packs/1,000 km^2^) to 1,800 km^2^ (wolf pack density  = 0.5 packs/1,000 km^2^). We distributed wolf territories in a regular pattern across the study area, assuming 8% overlap with neighbouring territories [6].

To simulate wolf pack movement following a fresh snowfall event, we first calculated wolf movement parameters including step length, turning angle, and number of travel moves from 2-hour wolf GPS locations. Following Turchin [27], we defined step length as the straight-line distance between two consecutive telemetry locations and turning angle as the change in direction between consecutive steps; number of travel moves (steps >150 m) was quantified over a 2-day period. Because the model proposed by Webb and Merrill [13] was created to survey wolf tracks in snow during winter, we only used GPS location data from December–February (i.e., months with consistent snow cover on the ground) for our simulations. We assessed differences in average step length between months and across years. We found no inter-annual variation in step length (*t* = 1.66, *P* = 0.09) and thus combined data from all years; however, wolf step lengths differed between winter months (*t_Jan-Feb_*  = 2.72, *P*<0.05; *t_Jan-Dec_*  = −3.13, *P*<0.05; *t_Feb-Dec_*  = −7.92, *P*<0.05), with wolf movements being on average shortest in February and longest in December (Table 1).

**Table 1:** Two-day movement parameters for 11 GPS-collared wolves in northeastern Alberta, Canada, 2006–2008. Parameters are based on 2-hour GPS locations.

Month	Step length (m)		Turning angle (°)	
	Mean	SD	Mean	SD
December	3,013	2,657	−2.46	103.85
January	2,118	2,159	2.56	108.17
February	1,488	1,606	0.48	105.12
Dec. to Feb.	1,928	2,074	0.10	105.12

Step length and turning angle distributions were used in a 2-state, habitat-biased correlated random walk movement model to move a wolf pack across the landscape for a 2-day interval following the steps described in Webb and Merrill [13]. The two movement states in the model were “moving” and “not moving”. Not moving states were identified as those steps <150 m, which are generally associated with bed-sites and kill-sites and lead to no net displacement [28]. The habitat bias in the model was derived using a resource selection function [29] developed for the WSAR study area using wolf GPS locations collected between October and March (i.e., snow-covered months; Appendix S1). Separate step length and turning angle distributions were developed for February and December (i.e., the complete range of wolf movement rates in our study) and used to simulate 1,000 wolf travel paths within each wolf pack territory for each of the two months. We verified that simulated movements closely replicated actual wolf movements by comparing total distance moved (km), and total number of road, pipeline and river crossings of 22 simulated and real 2-day paths, as paired by the start location of a real wolf path. Because we assumed that wolf pack territory sizes decreased as the density of wolf packs increased on the landscape, we also tested for an effect of changing wolf territory size on winter wolf movement rates. Winter wolf movement rates were not influenced by territory size based on a non-significant correlation between average winter 2-hour step length and winter 100% minimum convex polygons (*r_s_* = 0.23, *P* = 0.50, *n* = 11).

To simulate virtual wolf surveys, we randomly selected one 2-day movement path per wolf territory and then mimicked aerial surveys within 20% of randomly selected cells in each of the four survey grids. Occupancy was calculated as the proportion of survey units that intersected a wolf movement path. We iterated this virtual survey ten times at each wolf pack density and calculated the mean proportion of occupied survey units. We considered ten replicates per simulation to be adequate based on stabilization of the mean occupancy rate (Figure S1). For our virtual aerial surveys, we assumed that detectability of wolf paths within survey cells was 100%, irrespective of survey unit size. We discuss the implications of this assumption in the discussion.

We tested the sensitivity of the occupancy–abundance relationship obtained for each of four survey grids to variation in (1) wolf movement rates, (2) changes in pack cohesion (i.e., the number of individual hunting units within a territory), and (3) lone wolf movements. To depict variation in wolf movement rates, wolf travel paths were constructed by drawing 2-hour step lengths and turning angles from empirical distributions from December (long movements) versus February (short movements). Changes in pack cohesion were assessed at three levels: complete pack cohesion (i.e., all wolves followed the same movement path), two independent hunting units, and four independent hunting units. To simulate movements where each wolf pack was divided into two or four hunting units, we randomly selected two or four 2-day movement paths per wolf territory. We assessed the effect of lone wolves crossing the landscape by comparing simulations assuming no lone wolves with simulations assuming that 13% of the wolf population were loners [30]. To construct travel paths of lone wolves, we used the habitat-biased correlated random walk movement model described above but with the exception that we did not constrain movement paths to occur within the boundaries of a territory.

All statistical analyses were performed in R [31].

## Results

During the months of December, January and February, wolves in WSAR were actively moving during 57% ± 14% (mean ± SD; range  = 38%–82%) of the recorded 2-hour time steps and moved an average of 1,017 ± 595 m/hour. During December, the distribution of active step lengths followed a log-normal distribution (Figure S2) with a mean step length of 3,013 m (range  = 162 m–12,283 m; Table 1). Likewise, the distribution of active step lengths during February followed a log-normal distribution (Figure S2) with a mean step length of 1,488 m (range  = 151 m–11,843 m; Table 1). Two-day simulated wolf travel paths did not differ significantly from observed wolf paths in their total length, number of river or pipeline crossings, or in the number of survey units intersected in each of the four survey grids (Table 2).

**Table 2:** Characteristics of 22 real and simulated 2-day wolf travel paths in northeastern Alberta, Canada, 2006–2008.

Path characteristics	Real		Simulated		P-value
	Mean	SD	Mean	SD	
Path length (km)	15.49	10.61	18.75	5.89	0.21
No. survey units intersected					
36 km^2^ grid	2.55	1.47	3.18	1.59	0.13
144 km^2^ grid	1.82	1.14	1.73	0.94	0.72
400 km^2^ grid	1.32	0.57	1.36	0.73	0.67
576 km^2^ grid	1.27	0.63	1.27	0.63	1.00
No. road and/or pipeline intersections	2.95	4.45	5.45	6.26	0.07
No. of minor river intersections	4.55	4.89	6.45	5.05	0.11

Total length (km), number of survey units (from four different grids) intersected, number of minor river crossings, and number of road and pipeline intersections were compared used paired *t*-tests**.**

**Figure 1:**
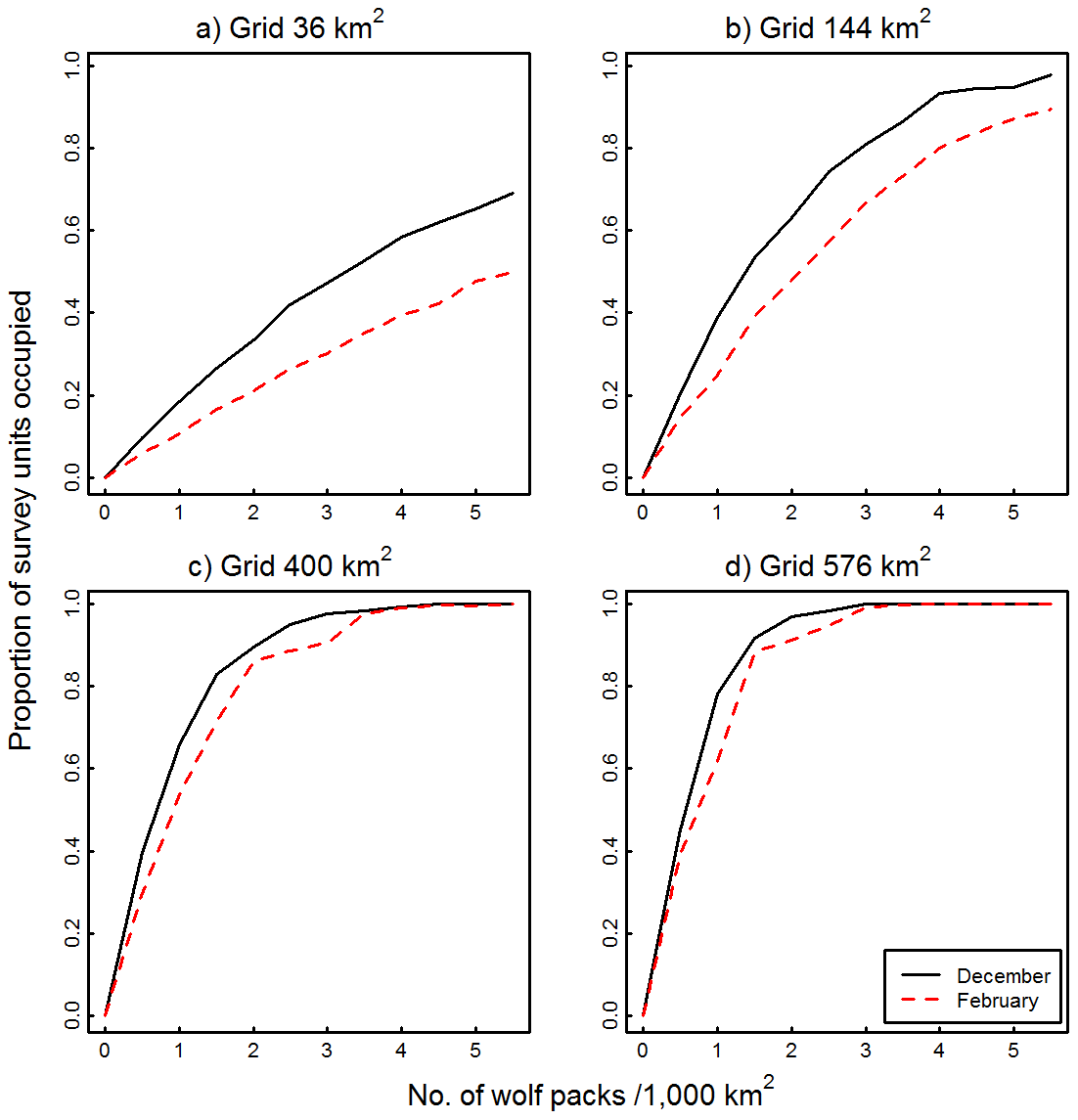
Effects of variation in wolf movement rate on occupancy–abundance relationships. Occupancy–abundance relationships were estimated from the proportion of survey units in which wolf tracks were detected during simulated aerial surveys as a function of wolf pack density (No. packs/1,000 km^2^). Wolf movements were based on February (short movements) or December (long movements) step length and turn angle distributions from 2-hour GPS locations from 11 wolves, northeastern Alberta, Canada, 2006–2008. Simulations were run for four grids: (a) 36 km^2^, (b) 144 km^2^, (c) 400 km^2^, and (d) 576 km^2^.

We found that the occupancy–abundance relationship estimated from the two smaller survey units (36 km^2^ and 144 km^2^) was sensitive to variation in wolf movement parameters (Figure 1a, b). For any given wolf pack density, occupancy rates were higher if wolf movements were long (Dec. movements) compared to short movements (Feb. movements). More importantly, the difference in occupancy rate increased as wolf pack density increased. Conversely, the relationship derived using the two larger survey units (400 km^2^ and 576 km^2^) was not as sensitive to variation in wolf movement parameters, with occupancy–abundance curves being similar over the range of wolf pack densities that we simulated (Figure 1c, d).

**Figure 2:**
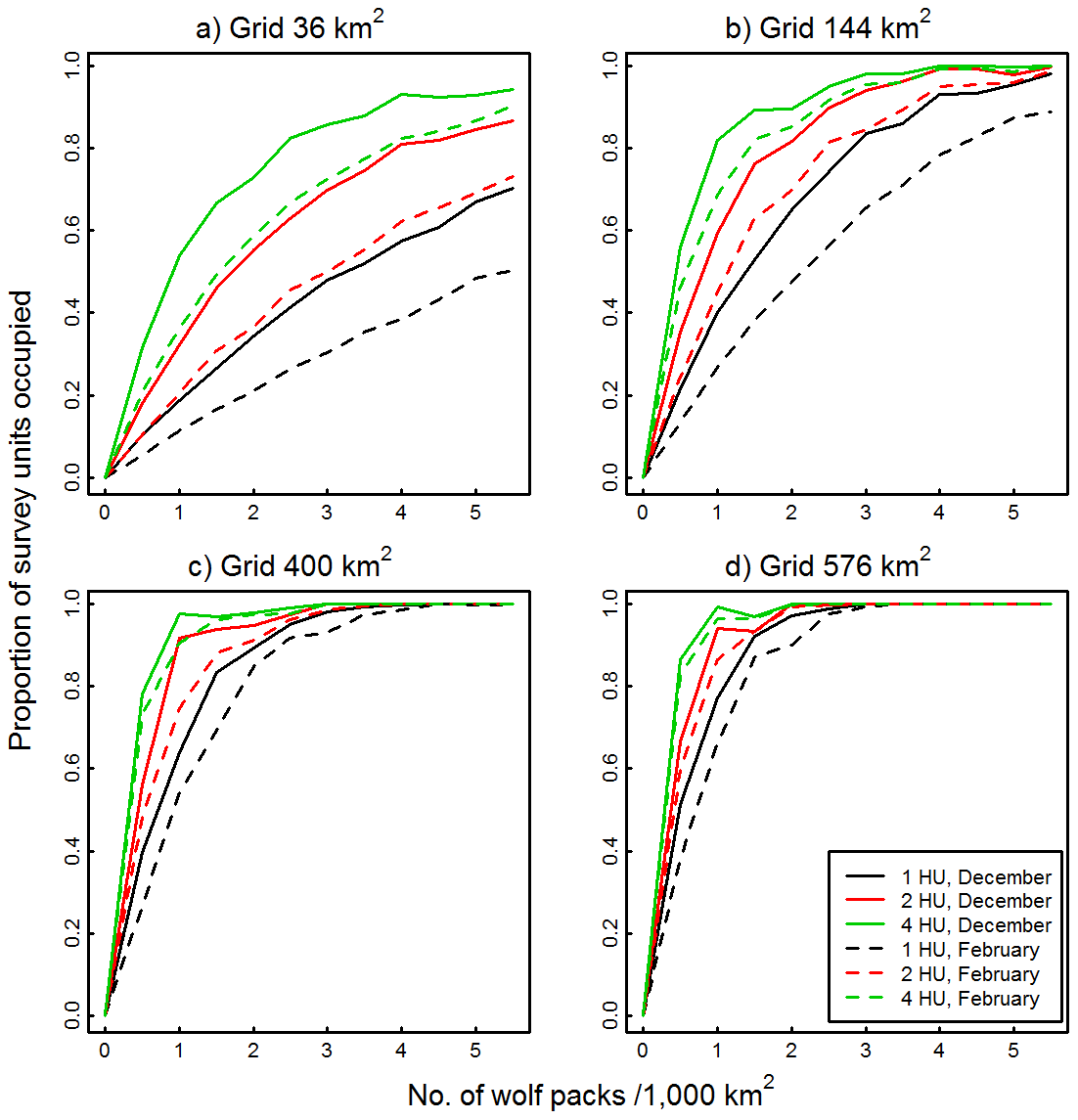
Effects of variation in wolf pack cohesion on occupancy–abundance relationships. Occupancy–abundance relationships were estimated from the proportion of survey units in which wolf tracks were detected during simulated aerial surveys as a function of wolf pack density (No. packs/1,000 km^2^). Movements of one, two, or four wolf hunting units (HU) were simulated using December or February step length and turn angle distributions from 2-hour GPS locations from 11 wolves, northeastern Alberta, Canada, 2006–2008. Simulations were run for four grids: (a) 36 km^2^, (b) 144 km^2^, (c) 400 km^2^, and (d) 576 km^2^.

The occupancy–abundance relationship estimated from all four survey unit sizes was sensitive to changes in wolf pack cohesion (Figure 2). Occupancy rates increased as the number of individual hunting units moving within a given territory increased from a cohesive pack to four independent units. Further, divergence in occupancy rate curves between the 3-levels of pack cohesion we simulated increased as wolf pack density increased. Comparatively, the two larger survey units (Figure 2c, d) were less sensitive to changes in pack cohesion than the two smaller survey units (Figure 2a, b). For example, the 36 km^2^ survey grid estimated that at a density of 1 wolf pack/1,000 km^2^ the occupancy rate would be ∼0.2 when wolf packs hunted as a cohesive unit, whereas it would be ∼0.6 when wolf packs split into 4 independent units, i.e., a 3-fold difference in occupancy rates (Figure 2a). Conversely, at that same wolf pack density the 576 km^2^ survey grid estimated an occupancy rate of 0.8 when wolf packs hunted as a cohesive unit and an occupancy rate of ∼1 when they split into 4 independent units, i.e., a smaller difference of 25% (Figure 2d). The sensitivity of the occupancy–abundance curves to changes in pack cohesion simulated using the empirical distribution from February (Figure 2) were very similar to those observed using the step length distribution from December.

**Figure 3:**
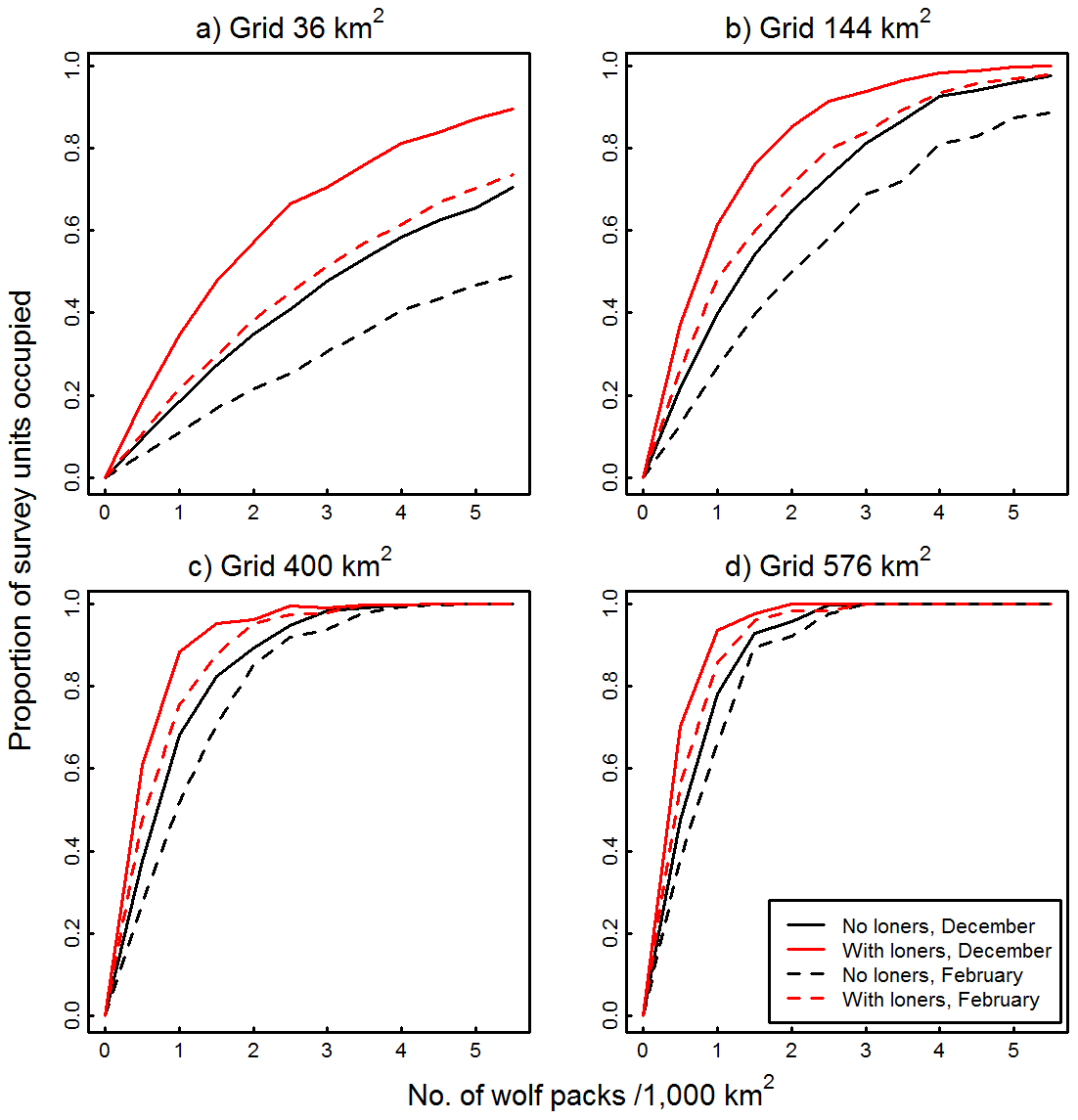
Effects of the presence of lone wolves on occupancy–abundance relationships. Occupancy-abundance relationships were estimated from the proportion of survey units in which wolf tracks were detected during simulated aerial surveys as a function of wolf pack density (No. packs/1,000 km^2^). Wolf movements were simulated using December or February step length and turn angle distributions from 2-hour GPS locations from 11 wolves, northeastern Alberta, Canada, 2006–2008. Lone wolves movements were simulated using the empirical distributions from December or February but without constraining travel paths to occur within territory boundaries. Simulations were run for four grids: (a) 36 km^2^, (b) 144 km^2^, (c) 400 km^2^, and (d) 576 km^2^.

All four grid sizes were sensitive to the inclusion of lone wolves (Figure 3). For the two smaller survey units, occupancy rates were higher if lone wolves were included in simulations, with divergence between the two curves being greatest at higher wolf pack densities (Figure 3a, b). This divergence was more pronounced for the 36 km^2^ survey units than for the 144 km^2^ units; in the latter grid the two curves converged at densities of ∼5.5 wolf packs/1,000 km^2^. Similarly, occupancy rates were higher if lone wolves were included in the simulations using 400 km^2^ and 576 km^2^ survey grids; however, divergence between the two curves was greatest at medium wolf densities. The sensitivity of the occupancy–abundance curves to the presence of lone wolves simulated using the empirical distribution from February (Figure 3) were very similar to those presented using December movements.

## Discussion

A parameterized occupancy–abundance relationship has high value for any wolf monitoring or management program if it can provide robust estimates of wolf abundance and if the relationship can be generalized to a number of different systems. However, deriving such a relationship empirically would be prohibitively expensive and time consuming. As an alternative, Webb and Merrill [13] introduced the idea of using an occupancy–abundance model developed from simulated track surveys to estimate wolf abundance in large areas of heavy forest cover where more accepted methods of estimating abundance might be unfeasible. Despite the obvious novelty and utility of the approach, our results show that estimates of wolf abundance derived in this way are highly sensitive to the size of the survey units (also see [14], [16]), as well as to the biology and behaviour of the species being surveyed.

We found that smaller survey units (36 km^2^ and 144 km^2^) were more sensitive to variation in wolf movement rates than larger survey units (400 km^2^ and 576 km^2^). As a consequence, estimates of occupancy, and thereby wolf pack density, will vary widely depending on distances traveled by wolf packs over the two days post snowfall event when snow-tracking–occupancy surveys generally occur. In our study, wolf movement rates were on average longest in December and shortest in February, probably reflecting responses to seasonal increases in air temperature and decreases in snow depth [32]. However, variation in movement rates can also occur at shorter time scales (i.e., daily as opposed to monthly) because of differences in travel strategies, such as intensive versus extensive territorial movements [33], [34]. Further, inter-annual variation in movement rates may also occur in response to differences in climatic conditions. In any case, the implication is that management decisions might differ depending on whether an occupancy survey is completed during a bout of long versus short distance movements.

Based on our simulations, increasing survey unit size from 36 km^2^ to 400 km^2^ or 576 km^2^ reduced the sensitivity of the occupancy–abundance relationship to variation in wolf movement rates. Large survey units are more likely to include the entirety of long and short movements, whereas small survey units will only be intersected by portions of a long wolf path, resulting in an estimated occupancy rate that is biased high. Sensitivity of occupancy–abundance relationships to the scale at which they were developed has been reported before [16], [17], [35]. Thus, it remains challenging to align the spatial distribution of a species with the appropriate scale at which to sample its occupancy.

Occupancy–abundance relationships were sensitive to changes in wolf pack cohesiveness, with occupancy rates being biased high when wolf packs were fragmented into more than one hunting unit. Whilst wolf packs are relatively cohesive in winter compared to summer, they can become less cohesive in winter as the breeding season (Feb.–Mar.) approaches [6], [36]. From a logistical perspective, ensuring that occupancy surveys are always conducted during the middle of winter, when packs are more likely to move as a cohesive unit [37], [38], should control for this potential source of bias. It should also be noted that the discrepancy between the estimated occupancy–abundance curves for one versus >1 hunting units was larger for surveys conducted within the smaller survey units than within the larger units. Movement paths created by independent hunting units from the same wolf pack were still constrained to occur within the territory boundaries of that pack, which in most cases fell within the same large survey cell. This result reinforces the fact that large survey units are less sensitive to changes in wolf pack cohesiveness.

Our final simulation showed that the presence of lone wolves can affect the estimation of abundance based on occupancy surveys, albeit small survey units were more sensitive to this factor than larger ones. This occurred because more independent wolf units moving throughout the survey grid resulted in an increased likelihood of wolf track–survey cell intersections. This effect was similar to that observed for pack cohesiveness; however, in this instance lone wolf movements were not restricted to occur within territory boundaries and thus were more likely to increase wolf track–survey cell intersection rates. As Webb and Merrill [13] state, this factor can be accounted for by using experienced observers that are able to distinguish tracks made by lone individuals from those made by the pack or sub-units of the pack (also see [11]). In this case, if only the tracks of lone animal were identified within a survey unit, the unit would be classified as ‘tracks absent’ for the estimation of occupancy rate. However, the observations of tracks of lone animals could be used to estimate an upper bound of wolf abundance.

The simulated occupancy–abundance relationship showed similar behaviour in response to changes in wolf pack cohesiveness or the presence of lone wolves regardless of whether long (Dec.) or short (Feb.) movement rates were used. This suggests that these factors affect the estimated relationship independently, as opposed to having interacting effects, and that each source of bias can be addressed separately. Further, it also shows that the model performs similarly regardless of the monthly data used to develop it, suggesting that abundance estimates would be comparable across surveys so long as they are derived consistently.

In our simulations, the occupancy–abundance relationship most robust to changes in wolf movement behaviour was that developed using the largest grid size (576 km^2^). We chose this size because it corresponded to the size of an average wolf territory in our area [8]. Other studies have also used estimated territory size of the species of interest to define occupancy survey unit size [39], [40]. The rationale behind this approach is that it complies with the assumption that there should be no (or negligible) movement of individuals between sampling units, at least during the period surveyed. In our study area, only three wolf pack territories fell completely within a single 576 km^2^ cell, whilst the remaining five territories overlapped 2–4 cells. Thus, although the entirety of the wolf territory might not fall within one single cell, the territory boundary still constrains the simulated wolf movements to occur within a single or a small number of cells; this was not be the case for the smaller-cell grids (e.g. wolf territory–cell intersections ranged from 4 to 26 for the 36 km^2^ grid). However, variation in wolf territory size is not uncommon, particularly between study areas [6], [21], which might complicate the task of determining the most appropriate scale at which occupancy should be measured. Conversely, Gopalaswamy et al. [35] showed that using a survey unit size sufficiently large to circumscribe expected daily movement of the study species provided good estimates of abundance. Wolves in our study area moved an average of 10 km per day during snow-covered months, a scale which corresponds to the 144 km^2^ grid. However, occupancy estimates from this survey grid were still very sensitive to wolf movement behaviour, probably reflecting the fact that daily wolf movements can range from 1 to 47 km and thus a large number of movements can still intersect more than one cell of this grid. Conversely, 97.5% of daily wolf movements were ≤ 25 km, i.e. approx. the scale of our 576 km^2^ grid, suggesting that most wolf movements were contained within a single cell and that is why the larger grid was robust to the changes in wolf movement behaviour that we simulated. Thus, as stressed by Gopalaswamy et al. [35], the importance of using reasonably accurate estimates of daily movement ranges to inform the design of occupancy–abundance surveys cannot be overstated.

Our simulations assumed that detection of wolf tracks in snow was 100% and that it was constant irrespective of survey unit size. Although it is likely that individual large units will need to be surveyed along longer flight paths than individual small units, we believe that other factors such as the amount of closed versus open habitat or weather conditions will influence detection probabilities to a larger extent than size [20]. Thus, the approach described here would greatly benefit from the explicit incorporation of variable detection rates based on cell size as well as other influential factors. Further, the length of the flight path per survey unit will need to be determined before conducting field surveys. This can be achieved using simulations (as described by Webb and Merrill [13]) and will likely be a trade-off between maximizing probability of detecting wolf tracks at an acceptable cost. In cases where funding is limited, the approach described by Karanth et al. [41], whereby survey efforts are reduced in cells with low proportion of habitat for the species of interest, can help alleviate this trade-off. Other alternative sampling protocols that could be adapted to the occupancy survey are illustrated in [42].

Occupancy–abundance relationships developed from larger survey units were more robust to changes in wolf movement rates, pack cohesion and the presence of lone wolves than those developed from smaller units. However, larger units showed a change from a somewhat linear occupancy–abundance relationship to an asymptotic one at high wolf pack densities. Asymptotes occurred when survey unit size was substantially larger than territory size, meaning that individual survey cells were likely to contain multiple packs and their movements. Consequently, the potential to obtain different wolf densities from similar occupancy rates arises, and thus the large survey unit model is incapable of accurately estimating wolf density at high occupancy rates. We suggest this model will reliably estimate wolf density at occupancy rates of approx. ≤0.8, but at higher occupancy rates this model should only be used to infer that the surveyed region has high wolf densities (in our example this would be >2 wolf packs/1,000 km^2^ or >15 wolves/1,000 km^2^). This information may be adequate for some management decisions such as reductions in wolf numbers for threatened woodland caribou conservation [43]–[45]. For example, wolf densities vary across their distributional range [6], with densities within woodland caribou distribution generally being low [30]. In this instance, Webb and Merrill's [13] approach, adjusted to an appropriate survey unit size, might allow managers to infer that wolves are at a density similar to that reported by Fuller and Keith [30], that they are above this threshold but below approx. 15 wolves/1,000 km^2^, or that densities are >15 wolves/1,000 km^2^. In the last instance, the need for wolf control – if this is the management objective – will be obvious despite the inability of the model to provide accurate wolf density estimates at high occupancy rates.

## Conclusions

Field occupancy surveys based on tracks seen in snow, combined with occupancy–abundance relationships developed from simulated wolf paths [13], can be a useful method to monitor wolves if biological knowledge of the species is incorporated in the design of such a sign survey. In particular, survey unit size should be increased to the approx. size of a wolf pack territory, the presence of lone wolves should be identified by experienced snow-trackers, and field surveys should be conducted at approx. the same time during mid-winter. Of these three requisites, obtaining accurate estimates of territory size will be most difficult. If an average territory size for the region of interest is unknown, then we recommend that wolf density estimates should be considered coarse (low, medium or high) and best suited for broad management purposes. Ideally, virtually-derived occupancy–abundance relationships should be empirically tested; however, this may be difficult and probably beyond most research budgets. Further, because the model described here is sensitive to variation in territory size at high occupancy rates, caution should be given to generalizing results to other study areas. In this instance, the most prudent course of action might be to restrict the approach to estimates of occupancy; that is, using occupancy rates to track changes in relative abundance. If surveys are designed to incorporate detectability, error associated with occupancy rates can be generated, further increasing the interpretative power associated with using occupancy as an index of long-term population trends. As is the case with any model that attempts to recreate the real-world, we showed that the approach suggested by Webb and Merrill [13] is sensitive to violations of its assumptions. Thus, we caution practitioners to be cognizant of the range of conditions under which the model is most likely to provide reliable measures of abundance. To broaden its applicability and reliability of the estimates, further modelling work could formally incorporate some of the biological factors into the model framework itself. Although occupancy surveys will remain susceptible to logistical factors such as tracking conditions and observer expertise, we suggest that carefully designed surveys coupled with virtually-derived occupancy–abundance relationships have the potential to provide cost-effective estimates of wolf abundance for broad regions.

## Supporting Information

Appendix S1 **Habitat bias in wolf movement modelling.**
(DOCX)

Figure S1 **Stabilization of the mean occupancy rates.** Mean occupancy rate is shown as a function of the number of simulations performed for each of four cell sizes and 11 wolf densities.
(TIFF)

Figure S2 **Step length distribution for (a) December and (b) February 2-hour movements by 11 GPS-collared wolves in Northeastern Alberta, Canada.**
(TIFF)
