# Supplementary material for: Can Occupancy–Abundance Models Be Used to Monitor Wolf Abundance?
Source: PLoS One. 2014 Jul 23;9(7):e102982. doi: 10.1371/journal.pone.0102982 (PMC4108393; doi:10.1371/journal.pone.0102982)
Supplement: Appendix S1 — Habitat bias in wolf movement modelling. (DOCX) [file pone.0102982.s001.docx]

**Appendix S1: Habitat bias in wolf movement modelling**

The habitat bias in the model was derived using a resource selection function (RSF; [1]) estimated from wolf GPS locations collected between Oct. and Mar. (i.e., snow-covered months; [2]). Characteristics of wolf GPS locations were compared with those at available points (1,000 / wolf pack) randomly located within the 100% Minimum Convex Polygon (MCP) home range of each pack. We developed models including fixed-effects for habitat covariates and a random intercept for each pack [3], and used Akaike Information Criterion (AIC) to identify the top model based on an a priori candidate set [4]. Covariates used to describe wolf habitat selection included 1) *landcover*: number of 25-m×25-m cell of conifer forest, deciduous forest, mixed forest, forestry cut-blocks of three age classes (young, middle, and old), fen and water bodies within 500-m; 2) *distance to features*: distance (km) to nearest road, seismic line, upland habitat edge, or minor river/stream, 3) density of anthropogenic linear features (km/km^2^) within 500-m; and 4) *prey distribution*: probability of occurrence for moose (*Alces alces*), white-tailed deer (*Odocoileus virginianus*), woodland caribou (*Rangifer tarandus caribou*), and beaver (*Castor canadensis*) derived from 5 fixed-winged aerial surveys conducted during the winters of 2004–2007 [5]. Data on anthropogenic and natural features were mapped from the Alberta Vegetation Inventory (AVI, [6]). Ungulate and beaver lodge sightings from winter aerial surveys were modelled using logistic regression [7] to estimate winter RSFs for each prey species separately, and the predicted probability of prey occurrence at each wolf location and random point were used in wolf RSF modelling [8].

Winter wolf resource selection function values were scaled between 0 and 1 by subtracting the lowest RSF value in the landscape from each cell, and dividing by the maximum RSF value in the landscape. We validated the top RSF model using k-fold cross validation [9]. The winter RSF surface predicting probability of wolf use was used to introduce a habitat-bias into the movement-based occupancy-abundance model as described by Webb and Merrill [10].

The RSF with lowest AIC included proportion of middle-aged cutblocks, deciduous, mixed-wood and conifer forest, fen and water (Table 1). Probability of wolf occurrence was positively related with the probability of moose occurrence. This model also included distance to minor river/stream and to upland habitat edge. Finally, it included an interaction between moose occurrence and water as well as between fen and distance to stream. The top model performed well as indicated by a mean Spearman’s rank correlation coefficient from 5-fold cross validation of 0.988 (p<0.01).

**REFERENCES**

1. Manly BFJ, McDonald LL, Thomas DL (2002) Resource selection by animals, statistical analysis and design for field studies. Nordrecht, The Netherlands: Kluwer. 192 p.

2. Latham ADM (2009) Wolf ecology and caribou-primary prey-wolf spatial relationships in low productivity peatland complexes in northeastern Alberta. Dissertation, University of Alberta, Edmonton, Canada.

3. Hebblewhite M, Merrill EH (2008) Modelling wildlife–human relationships for social species with mixed-effects resource selection models. J Appl Ecol 45: 834–844.

4. Burnham KP, Anderson DR (2002) Model selection and multimodel inference: a practical information-theoretic approach. 2^nd^ Ed. New York: Springer-Verlag. 488 p.

5. Latham ADM, Latham MC, McCutchen NA, Boutin S (2011) Invading white-tailed deer change wolf-caribou dynamics in northeastern Alberta. J Wildl Manage 75: 204–212.

6. Nesby R (1997) Alberta vegetation inventory. Final version 2.2. Edmonton, Alberta, Canada: Alberta Environmental Protection.

7. Hosmer DW, Lemeshow S (2000) Applied logistic regression. 2nd Ed. New York: Wiley. 392 p.

8. Latham ADM, Latham MC, Knopff KH, Hebblewhite M, Boutin S (2013). Wolves, white-tailed deer, and beaver: implications of seasonal prey switching for woodland caribou declines. Ecography 36: 1276–1290.

9. Boyce MS, Vernier PR, Nielsen SE, Schmiegelow FKA (2002) Evaluating resource selection functions. Ecol Model 157: 281–300.

10. Webb NF, Merrill EH (2012) Simulating carnivore movements: an occupancy–abundance relationship for surveying wolves. Wildl Soc Bull 36: 240–247.

| **Table S1:** Best mixed-effects logistic regression resource selection model for wolves in the snow season (Oct. to Mar.) in northeastern Alberta, Canada, January 2006 to January 2008 (*n* = 5,244). | | | |
| --- | --- | --- | --- |
| Variable | β | SE | *P-value* |
| Cutblocks (6–30-yr old) | 0.907 | 0.292 | 0.002 |
| Deciduous forest | 0.303 | 0.132 | 0.022 |
| Fen | 0.439 | 0.170 | 0.010 |
| Mixed-wood forest | 2.392 | 0.194 | <0.001 |
| Upland conifer | 1.929 | 0.158 | <0.001 |
| Water | –5.748 | 0.516 | <0.001 |
| Distance to minor river/stream | –0.00049 | 0.00004 | <0.001 |
| Distance to upland habitat edge | –0.00011 | 0.00002 | <0.001 |
| Moose | 155.2 | 1,058.0 | 0.883 |
| Distance to stream × fen | 0.00131 | 0.00012 | <0.001 |
| Moose × water | 111,400.0 | 7,142.0 | <0.001 |
