# Supplementary figures and images for: Can Occupancy–Abundance Models Be Used to Monitor Wolf Abundance?
Source: PLoS One. 2014 Jul 23;9(7):e102982. doi: 10.1371/journal.pone.0102982 (PMC4108393; doi:10.1371/journal.pone.0102982)

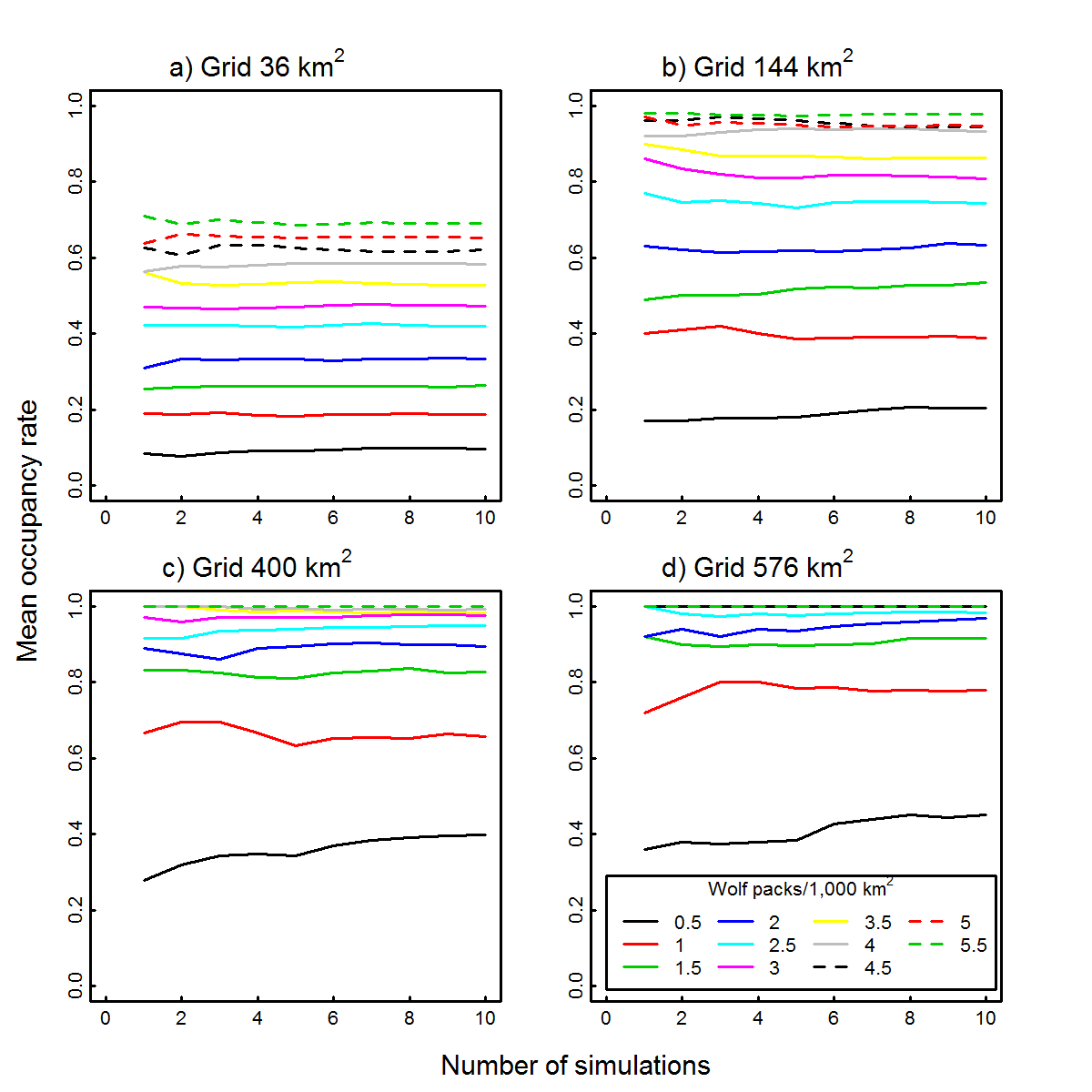

Supplement: Figure S1 — Stabilization of the mean occupancy rates. Mean occupancy rate is shown as a function of the number of simulations performed for each of four cell sizes and 11 wolf densities. (TIFF) [file pone.0102982.s002.tiff]

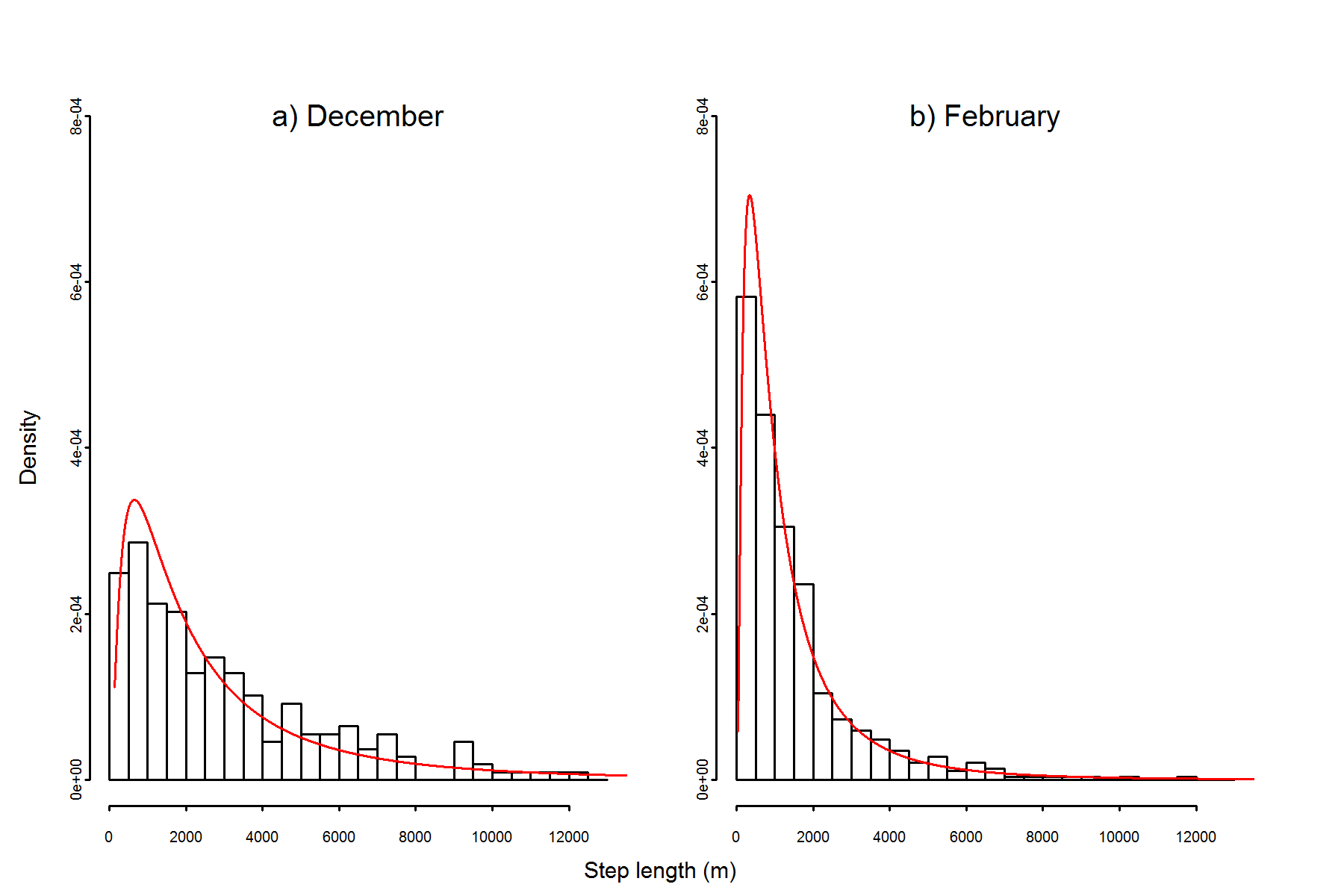

Supplement: Figure S2 — Step length distribution for (a) December and (b) February 2-hour movements by 11 GPS-collared wolves in Northeastern Alberta, Canada. (TIFF) [file pone.0102982.s003.tiff]
